# A systematic screening to identify *de novo* mutations causing sporadic early-onset Parkinson's disease

**DOI:** 10.1093/hmg/ddv376

**Published:** 2015-09-11

**Authors:** Celia Kun-Rodrigues, Christos Ganos, Rita Guerreiro, Susanne A. Schneider, Claudia Schulte, Suzanne Lesage, Lee Darwent, Peter Holmans, Andrew Singleton, International Parkinson's Disease Genomics Consortium (IPDGC), Kailash Bhatia, Jose Bras

**Affiliations:** 1 Department of Molecular Neuroscience, UCL Institute of Neurology, London WC1N 3AR, UK,; 2 Department of Neurology, University Medical Center Hamburg-Eppendorf (UKE), Hamburg 20246, Germany,; 3 Sobell Department of Motor Neuroscience and Movement Disorders, UCL Institute of Neurology, University College London, London WC1N 3BG, UK,; 4 Department of Neurology, University Hospital Schleswig Holstein, Campus Kiel 24105, Germany,; 5 German Center for Neurodegenerative Diseases, Tübingen, Germany,; 6 Department of Neurodegenerative Diseases, Hertie Institute for Clinical Brain Research, University of Tübingen, Tübingen 72076, Germany,; 7 INSERM U M27, Pitié-Salpêtrière Hospital, Brain and Spinal Cord Institute (ICM), Paris 75013, France,; 8 Medical Research Council Centre for Neuropsychiatric Genetics and Genomics, Institute of Psychological Medicine and Clinical Neurosciences, Cardiff University, Cardiff CF24 4HQ, UK and; 9 Laboratory of Neurogenetics, National Institutes on Aging, National Institutes of Health, Bethesda, MD 20892, USA

## Abstract

Despite the many advances in our understanding of the genetic basis of Mendelian forms of Parkinson's disease (PD), a large number of early-onset cases still remain to be explained. Many of these cases, present with a form of disease that is identical to that underlined by genetic causes, but do not have mutations in any of the currently known disease-causing genes. Here, we hypothesized that *de novo* mutations may account for a proportion of these early-onset, sporadic cases. We performed exome sequencing in full parent–child trios where the proband presents with typical PD to unequivocally identify *de novo* mutations. This approach allows us to test all genes in the genome in an unbiased manner. We have identified and confirmed 20 coding *de novo* mutations in 21 trios. We have used publicly available population genetic data to compare variant frequencies and our independent in-house dataset of exome sequencing in PD (with over 1200 cases) to identify additional variants in the same genes. Of the genes identified to carry *de novo* mutations, *PTEN*, *VAPB* and *ASNA1* are supported by various sources of data to be involved in PD. We show that these genes are reported to be within a protein–protein interaction network with PD genes and that they contain additional rare, case-specific, mutations in our independent cohort of PD cases. Our results support the involvement of these three genes in PD and suggest that testing for *de novo* mutations in sporadic disease may aid in the identification of novel disease-causing genes.

## Introduction

Although the detailed aetiology of Parkinson's disease (PD) remains largely unknown, data suggest the disease may be triggered through different mechanisms: protein inclusions accumulation, diminished mitochondrial activity, proteasomal/lysosomal dysfunction and impaired dopamine production (1). An increasing number of publications show a strong genetic component for PD. Studies in familial forms of PD have allowed for the identification of disease-causing mutations in several genes, causing either dominant or recessive forms of disease inheritance. Genome-wide association studies (GWAS) have also significantly contributed to a more comprehensive knowledge of the risk loci involved in PD (2). Despite these results, there is still a large number of sporadic, early-onset cases that carry no mutation in the known PD genes. These individuals have, in many cases, a form of disease that is indistinguishable from genetically linked disease.

*De novo* mutations have been commonly studied in neurodevelopmental disorders such as autism (3–5) and schizophrenia (6,7) with only a few examples in neurodegenerative diseases. *De novo* mutations in the *ATP1A3* gene have been found as the cause for rapid-onset dystonia parkinsonism (8), while Rosewich *et al.* (9) reported *de novo* mutations in this gene in alternating hemiplegia of childhood. De Carvalho Aguiar and colleagues (10) showed one *de novo* mutation in *TOR1A* in a primary torsion dystonia case. Two cases of static encephalopathy of childhood with neurodegeneration in adulthood showed mutations in *WDR45* (11). In all these cases, patients start to show symptoms during childhood or adolescence.

Recently studies have suggested the involvement of *de novo* mutations in other neurodegenerative diseases, such as in early-onset Alzheimer's disease (AD) (12,13) and amyotrophic lateral sclerosis (ALS) (14–17).

There is also preliminary evidence supporting the role of *de novo* mutations in PD: Puschmann and colleagues (18) reported on a presumably *de novo* A53T mutation in *SNCA* and non-mendelian multiplications of the gene (19,20) have also been shown. More recently, Hansen and collaborators (21) described two mutations in the *SLC6A3* gene (encoding the dopamine transporter, DAT1), one of which was presumed to be *de novo*. It should be noted however that in both the *SNCA* A53T and the *SLC6A3* cases, the authors were not able to positively confirm the presence of *de novo* mutations, given the absence of parental DNA samples.

Here, we hypothesize that a subset of early-onset PD cases, with no mutations in any of the known PD-causing genes, may be due to the occurrence of *de novo* mutations. We used whole-exome sequencing in full parent–child PD trios to unequivocally identify these events.

## Results

**Figure 1.**
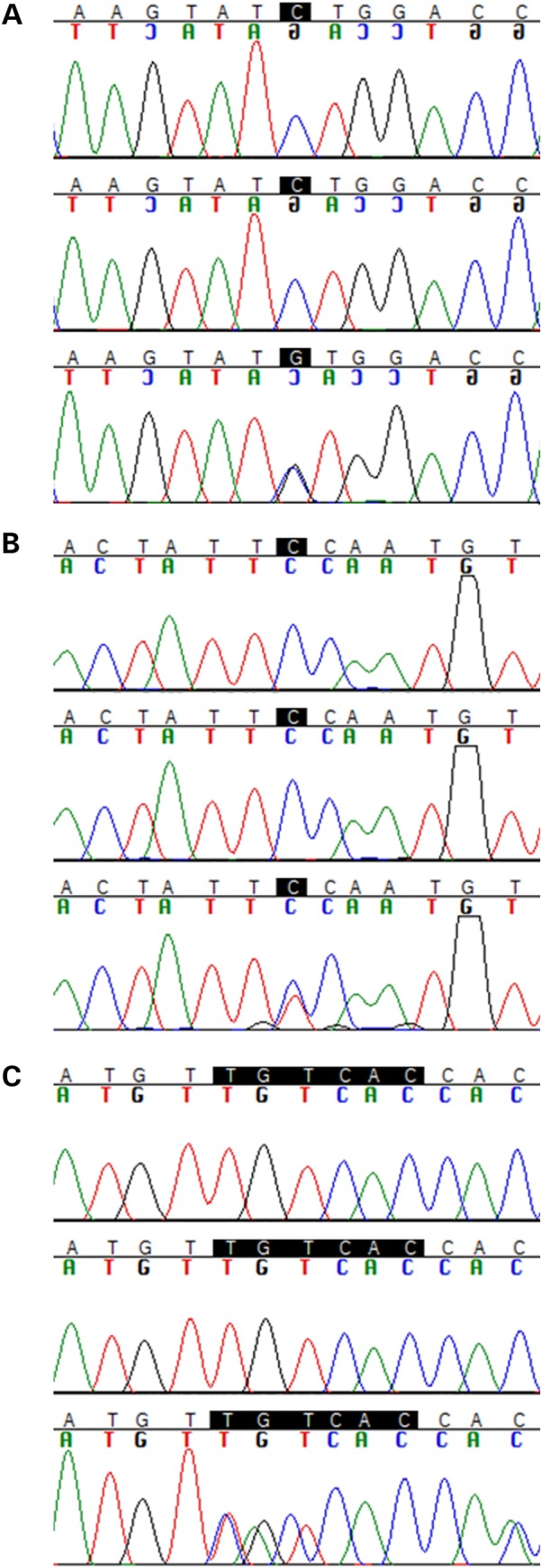
Sanger sequencing chromatograms for (**A**) *ASNA1* (p.L303V); (**B**) *PTEN* (p.P204S); and (**C**) *VAPB* (p.ΔV25). Top: father; middle: mother; bottom: proband.

From the systematic analysis of the whole-exome sequencing data, 24 genes showed *de novo* mutations in the trios. We validated 20 of the 24 variants with Sanger sequencing methods (Table 1) comparing sequences from both parents with the proband (examples for the three genes of interest in Fig. 1). The four variants not validated were false positives from the exome sequencing. We have identified, on average, one *de novo* coding event per trio, which is in line with what would be expected for the human population (22) (Table 2). Only four of the variants identified have been previously described in population databases: the variants in *EPPK1*, *COL12A1*, *PEPD* and *SLC52A1* have low frequencies ranging from 0.00003 to 0.003 in Exome Aggregation Consortium (ExAC). All other variants are presumably novel as they are absent from tested databases. PD cases did not show any potential pathogenic mutations in the known PD genes—all variants identified in those genes were either frequent in the general population, present in the unaffected parents, located in not conserved residues or predicted to be benign by PolyPhen and SIFT (23,24).

**Table 1.** List of *de novo* variants identified in the PD trios

Trio	Gene	Chromosome	Position	Transcript	Codon change	Amino acid change	Reference SNP	SIFT prediction	Polyphen prediction	CADD phred score	ESP6500	1000 Genomes	ExAC
1	COL12A1	6	75 887 555	NM_004370	cGa/cAa	R754Q	rs377480187	None	Probably_Damaging	32	0.000084	0	0.00003311
	RUNDC3A	17	42 390 571	NM_001144825	tGt/tTt	C108F		Deleterious	Probably_Damaging	22.2	0	0	0
	VAPB	20	56 9932 80	NM_004738	gaTGTt/gat	ΔV25		None	None	None	0	0	0
2	ANKRD13A	12	1 104 655 60	NM_033121	Ggt/Tgt	G312C		Deleterious	Possibly_Damaging	22.8	0	0	0
	MKS1	17	56 293 486	NM_001165927	aCc/aTc	T117I		Deleterious	Probably_Damaging	33	0	0	0
3	PAPD4	5	78 938 703	NM_173797	Tta/Gta	L241V		Tolerated	Benign	15.53	0	0	0
	VPS53	17	455 114	NM_001128159	Aag/Gag	K622E		Deleterious	Probably_Damaging	29.7	0	0	0
4	PMEL	12	56 349 087	NM_001200054	atG/atA	M614I		Tolerated	Benign	10.33	0	0	0
	SLC5A9	1	48 695 007	NM_001135181	cCt/cTt	P152L		Deleterious	Probably_Damaging	33	0	0	0
5	ASNA1	19	12 858 398	NM_004317	Ctg/Gtg	L303V		Deleterious	Benign	None	0	0	0
6	EPPK1	8	14 494 1903	NM_031308	aCg/aTg	T1840M	rs79961029	Tolerated	Benign	9.541	0.008705	0.00838658	0.003023
7	FBXL17	5	10 770 3586	NM_001163315	gaC/gaA	D354E		Tolerated	Possibly_Damaging	17.45	0	0	0
8	KCNV2	9	27 189 66	NM_133497	caG/caT	Q409H		Tolerated	Benign	13.86	0	0	0
9	LCT	2	13 654 8243	NM_002299	Aat/Cat	N1774H		Deleterious	Probably_Damaging	21.9	0	0	0
10	MGA	15	41 988 608	NM_001164273	cCa/cAa	P467Q		Deleterious	Probably_Damaging	16.87	0	0	0
11	PEPD	19	33 878 830	NM_000285	cGc/cAc	R437H	rs373297406	Tolerated	Benign	13.91	0.000329	0.000199681	0.00006709
12	PML	15	74 327 974	NM_033250	cgG/cgC	R676R		None	None	None	0	0	0
13	PSD4	2	11 395 0082	NM_012455	aAc/aGc	N585S		Tolerated	Benign	9.504	0	0	0
14	PTEN	10	89 711 992	NM_000314	Cca/Tca	P204S		Tolerated	Possibly_Damaging	32	0	0	0
15	SLC52A1	17	49 363 59	NM_001104577	Gcc/Acc	A414T	rs142353672	Tolerated	Benign	11.56	0.007	0	0.00005776

**Table 2.** Sequencing metrics

Proband	Total on target (bp)	Mean on target coverage	Fraction targeted bases >2x	Total *de novo* coding mutations
1	1 445 598 290	27.12	0.97	3
2	1 421 276 651	26.52	0.97	2
3	1 146 375 416	21.47	0.97	2
4	7 152 790 854	135.14	0.94	2
5	8 974 607 696	167.43	0.94	1
6	7 933 070 487	147.95	0.94	1
7	1 376 003 145	26.44	0.97	1
8	1 914 732 654	36.21	0.98	1
9	4 286 967 024	76.61	0.95	1
10	6 133 197 922	112.73	0.97	1
11	3 381 480 354	61.73	0.94	1
12	6 529 240 671	121.95	0.94	1
13	4 485 511 060	87.10	0.99	1
14	1 843 089 171	33.41	0.98	1
15	6 364 816 268	119.29	0.94	1
16	3 347 839 519	59.15	0.95	0
17	1 171 269 433	22.19	0.96	0
18	2 110 891 218	39.22	0.98	0
19	4 145 185 493	80.55	0.99	0
20	3 649 693 000	70.18	0.99	0
21	1 461 050 157	28.87	0.93	0
Average	3 822 604 118	71.49	0.96	0.95

**Figure 2.**
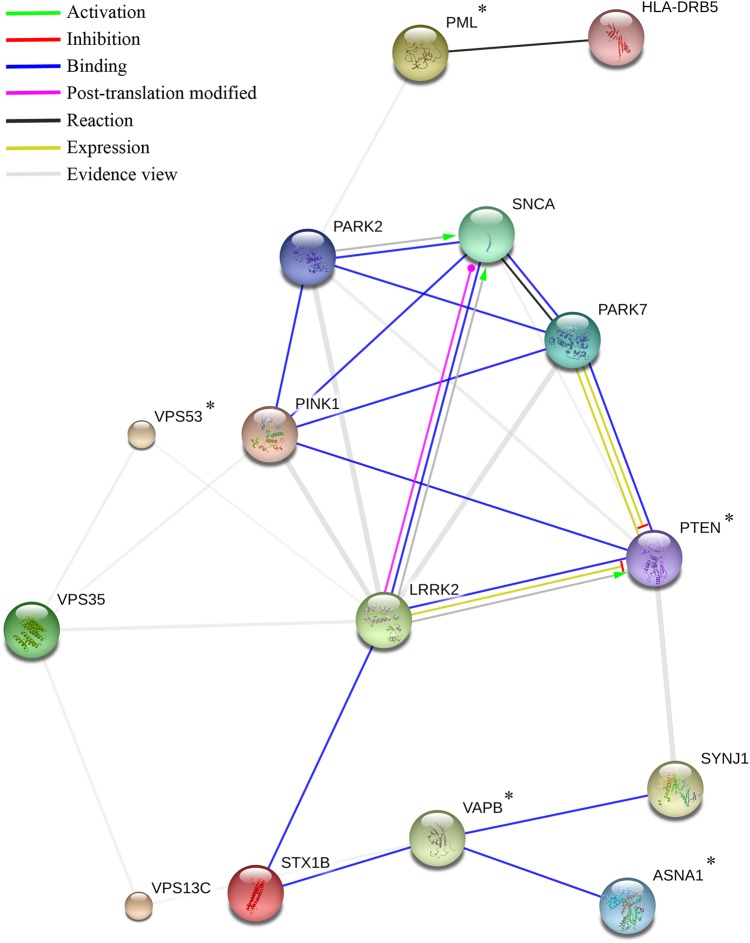
Action view from the STRING network analysis. Colours correspond to interactions according to the legend (top left); Evidence view (grey) corresponds mainly to interactions obtained from text-mining sources. The remaining interaction colours are based on published experimental results, with Green representing activation, Red inhibition, etc. If the directionality of the effect is known, this is indicated by the symbol at the end of the edge next to the protein that is acted upon. Down-regulation is a red bar and up-regulation is a green arrow. *denotes genes identified to carry *de novo* mutations.

From the protein network analysis of the 20 validated *de novo* mutation carrying genes, *PTEN*, *VAPB*, *ASNA1*, *PML* and *VPS53* showed interactions with the known PD genes (Fig. 2). However, *VPS53* and *PML* did not show high interaction scores with disease-causing genes. Robust functional interactions with known PD genes were identified for the three remaining candidates. (Table 3 and Fig. 2).

**Table 3.** Scores from the STRING protein–protein analysis restricted to *Homo sapiens*

Gene	Interaction	Total score	Co-expression	Experimental	Knowledge	Text-mining
PTEN	PARK7	0.990	0	0.844	0	0.940
	SYNJ1	0.928	0	0.116	0.900	0.242
	PINK1	0.699	0	0.172	0	0.643
	LRRK2	0.667	0	0.295	0	0.537
	PARK2	0.608	0	0	0	0.599
	SNCA	0.472	0	0	0	0.472
VAPB	STX1B	0.919	0	0.730	0	0.697
	SYNJ1	0.510	0	0.363	0	0.245
	VPS13C	0.449	0	0	0	0.436
PML	HLA-DRB5	0.900	0	0	0.900	0
	PARK2	0.507	0	0	0	0.507
ASNA1	VAPB	0.887	0	0.413	0	0.812
VPS53	VPS35	0.502	0.111	0	0	0.451
	LRRK2	0.413	0	0.102	0	0.374

The total scores are computed by combining the probabilities from the different evidence channels (co-expression, experimental, knowledge, text-mining), correcting for the probability of randomly observing an interaction, as described in STRING′s documentation.

A total of 10 interactions were observed between proteins corresponding to genes containing *de novo* variants and proteins corresponding to known PD genes. This is significantly higher than expected by chance (*P* = 0.009), however, after correcting for the total numbers of interactions observed for each protein, the result does not retain statistical significance (*P* = 0.162). Five out of the ten interactions involved *PTEN*. Nevertheless, this number was not significantly higher than expected by chance (*P* = 0.226), after correcting for the large total number of interactions involving *PTEN*.

Seventy-four interactions were observed between the proteins corresponding to the 33 known PD genes. This is significantly higher than expected by chance (*P* < 0.000001). Several individual proteins showed a significant excess of interactions, even after correcting for the multiple testing of proteins. The interaction network between the proteins corresponding to PD genes is shown in Supplementary Material (see Supplementary Material, Table S1 and Fig. S1).

Interestingly, when taking into account the allele frequency from the ExAC database for all the genes studied here, we observed the lowest total number of variants normalized by protein size to be present in the three genes with higher interaction with known PD genes—*PTEN*, *VAPB* and *ASNA1* (see Supplementary Material, Fig. S2), suggesting these may be under stronger selection pressure.

The International Parkinson's Disease Genomics Consortium (IPDGC) dataset shows 2 missense and 1 frameshift mutations in *PTEN* (all in PD cases, no rare variants were found in controls); 4 missense, 1 frameshift and 1 stop-gained mutations in *ASNA1*; and 5 missense and 1 deletion mutations in *VAPB*. With the exception of three common variants in *VAPB*, all others are exceedingly rare in the population (Table 4) and heterozygous in the IPDGC dataset.

**Table 4.** List of IPDGC variants in the top candidate genes

Gene	Chromosome	Position	rs_ids	Codon change	Amino acid change	MAF cases	MAF controls	MAF ESP6500	MAF ExAC	ExAC alleles
ASNA1	19	12 848 341		Frameshift (insG)	−8	0.039	0	0	0	0
		12 848 341		Tgg/Ggg	W8G	0.039	0	0	0	0
		12 848 342	rs138730527	tGg/tAg	W8* (stop gained)	0	0.099	0.0154	0.00001726	1
		12 849 344		Ctc/Gtc	L61V	0.039	0	0	0	0
		12 849 356		Cgt/Tgt	R65C	0.039	0	0	0	0
		12 858 023		Ctg/Gtg	L208V	0.039	0	0	0	0
		12 858 901	rs200489378	Ccc/Tcc	P344S	0.039	0	0.0077	0.00007819	9
PTEN	10	89 685 310		Aat/Gat	N69D	0.04	0	0	0	0
		89 692 866		aAt/aGt	N117S	0.039	0	0	0.000008237	1
		89 720 798		Frameshift (delTACT)	−317	0.039	0	0	0	0
VAPB	20	56 964 571		cGa/cTa	R19L	0.041	0	0	0	0
		57 014 058		Ttg/Atg	L125M	0.039	0	0	0.00001661	2
		57 014 075	rs146459055	gaT/gaG	D130E	0.079	0	0.0692	0.001359	163
		57 016 039		agttct/agt (del)	SS160S	0.313	0.298	0	0.001695	203
		57 016 076	rs143144050	atG/atA	M170I	0.313	0.099	0.1153	0.001373	165
		57 016 117	rs145483046	cGg/cAg	R184Q	0.039	0	0.0308	0.00007541	9

Synonymous mutations were not included.

MAF, minor allele frequency.

The frameshift mutation in *PTEN* (NM_000314:c.955_958delACTT:p.Thr319*) was identified in a case from the IPDGC dataset with positive, albeit limited, family history for PD: the proband's father developed the disease in his 50s. Samples from both parents and unaffected sibling were available. After testing for segregation in the limited family, we observed that the mutation in *PTEN* is, in all likelihood, *de novo*, since it is absent from either parent, and thus unlikely to cause the disease in this family. However, we should note that the age at onset is noticeably different between the affected father and the proband in this family (54 years in the father and 38 years in the proband).

When looking at the cases with confirmed *de novo* mutations in the genes with a functional interaction with known PD causing genes, these showed a typical presentation of PD.

The *PTEN de novo* mutation carrier, aged 23, is a British Caucasian male who noticed a left-sided thumb action tremor. Gradually, the tremor progressed to be present at rest and to involve his left arm. Four years later, he developed gait difficulties with some shuffling and stumbling. During the same period the tremor spread to involve his right arm as well, and at the age of 28 he was diagnosed with young-onset Parkinson's disease (YOPD). He was started on carbidopa/levodopa with significant improvement on his gait but no effect on the tremor. Only two years after treatment, he developed drug-induced dyskinesias. At the time his treatment had consisted of carbidopa/levodopa, rasagiline and ropinirole, and, thus, amantadine was added. Age 32 and due to clinical progression and particularly more severe dyskinesias, the patient underwent bilateral deep brain stimulation of the subthalamic nucleus with very good response. Currently, at the age of 36, he suffers from off-periods, has difficulties speaking, as well as increased urinary urgency. His medication consists of carbidopa/levodopa, entacapone, rasagiline, ropinirole and amantadine.

The *VAPB de novo* mutation carrier is a 44-year-old Caucasian man of British descent, who first noticed a left arm rest tremor at the age of 41. He also complained of shoulder stiffness on the same side and some difficulties in performing fine tasks with his left hand. Clinical examination revealed asymmetric parkinsonism (left>right side) and a subsequent dopamine transporter SPECT scan (DaTSCAN) confirmed the presence of a presynaptic dopaminergic deficit corresponding to the more affected body side. He was diagnosed with YOPD. Treatment was commenced with rasagiline and subsequently pramipexole was added.

The *ASNA1 de novo* mutation carrier developed PD at an age of 40 years. The first symptom was reduced right arm swing, soon followed by tremor, muscle stiffness, dexterity, gait and writing difficulties. At age 43 he experienced fatigue, constipation and freezing. The father is healthy and the mother suffers from rheumatoid arthritis. The family history for PD is negative.

## Discussion

This is the first study to systematically screen for *de novo* mutations in early-onset sporadic PD using parent–proband trios. We performed exome sequencing in 21 trios and found 20 *de novo* mutations (19 single nucleotide variants and one in-frame deletion). All probands were heterozygous in those positions. The genes screened are involved in a variety of functions from poly(A) polymerase activity to ubiquitin-binding protein (see Supplementary Material, Table S2) and some are thought to be involved in known PD pathways.

*PTEN*, the phosphatase and tensin gene, has functions in neuronal migration, neuron number regulation and apoptosis in response to oxidative stress (25). *PTEN* somatic down-regulation has been linked to tumours, neural proliferation and is being proposed as a target for neuroprotection in PD cases (26,27), whereas *PTEN* mutations in the germline have been linked to developmental neurological diseases (28). Mutations in *PTEN* are mainly linked to macrocephaly, autism and ataxia (29–32).

We found novel variants in *PTEN*. One of our trio-based proband showed a *de novo* missense mutation in *PTEN* (p.P204S). Interestingly, mutations in *PTEN* seem to be rare: there is only one stop-gained and one frameshift in ExAC for a total of 149 coding variant alleles out of ∼120 000. Similarly, in our IPDGC dataset we found a relatively small number of variants in *PTEN*: four synonymous mutations, two missense mutations (p.N69D and p.N117S) and one frameshift, all of them very rare or non-existent in the population (Table 4). The two missense mutations are from patients with sporadic PD and the patient with the frameshift mutation has a reported positive familial history of PD. Although the frameshift mutation did not show segregation in this family, it is remarkable that it is another apparent *de novo* mutation in a case with a much lower age at onset than the affected father.

The delicate equilibrium of the various functions of *PTEN* means that it is often difficult to interpret genetic variability in this gene. *PTEN* transcriptionally activates *PINK1*, which is downregulated in the event of *PTEN* ablation (33,34). *PINK1* variants are responsible for PD through a mechanism that is thought to involve oxidative stress caused by mitochondrial dysfunction. *PTEN* was also linked to the induction of cytochrome c oxidase activity and ATP production in mitochondria (34). On the other hand, *PTEN* induces cell death and inhibits the PI3K/Akt signalling that reduces inflammation and promotes cell proliferation and survival (26). Furthermore, *DJ-1* physically binds to *PTEN* negatively regulating its activity which, in turn, upregulates PI3K/Akt pathway (35).

*PTEN* has a large range of activities and cellular locations in a variety of cells. Different mutations in this gene can affect PTEN activity and localization in different ways (36). Deletion of the N-terminal domain PBM (residues 1–15) impedes PTEN interaction with the plasma membrane [see (37) for PTEN protein structure]. When the phosphatase domain (residues 15–185) is mutated, there is a reduction in phosphatase activity and an increase in PI3K/Akt activity. The C2 domain (residues 185–351) inhibits cell migration. The C-tail domain (residues 351–401) is important for interactions with transmembrane proteins and a 2 bp C-terminal PDZ-binding domain (36).

Most of the mutations described for developmental anomalies are missense mutations and appear mainly on the phosphatase and C2 domains, e.g. (30,32,38,39). In addition, a variant can affect the gene's function only in specific subcellular localizations. In 2007 Trotman and colleagues (40) described a missense mutation p.K289E that did not affect the membrane localization of PTEN, but the protein was not present in the nucleus. Another nuclear mislocalization was apparent when mutations were present in the ATP binding motifs at the residues 60–73 and 122–136 (41). This nuclear mislocalization was observed in AD cases with the redistribution of PTEN to the cytoplasm into intracellular neurofibrillary tangles and senile plaques (42).

VAPB is involved in vesicular and endosomal trafficking and was identified in ALS cases (43–45). *VAPB* expression levels are significantly lower in the spinal cord of ALS cases (46). Furthermore, missense mutations in *VAPB* protein such as p.P56S and p.T46I cause intracellular ubiquitinated aggregates that predominantly affect motor neurons and induce motor neuron death (43,45).

We found a *de novo* in-frame deletion of a valine residue at position 25 (p.ΔV25) in *VAPB*. Only one other deletion in *VAPB* was documented, p.ΔS160, which is common in the population and any association with ALS was discarded (47). Interestingly, there are only two high impact variants in *VAPB* in the ExAC dataset: two nonsense variants each in a single sample, out of >60 000 individuals.

Structural studies indicate human VAPB protein is organized in three main conserved domains consisting of the N-terminal major sperm protein (MSP) domain from residue 1 to 125, the coiled coil (CC) domain from residue 151 to 195 and the C-terminal transmembrane (TM) domain including the remaining residues until residue 243 (48). The MSP is very important in VAPB as it is the domain that binds to the FFAT (two phenylalanines in an acidic tract) motif of lipid-binding-proteins and is involved in the ubiquitination pathway. VAPB binds through that domain to proteins such as *STX1A* and STX1B, p97 ATPase and *ASNA1* (49). Misfolding of MSP domain induces the formation of the aggregates and the binding site becomes unavailable on the endoplasmic reticulum (50).

As Gupta *et al.* (48) noticed, the p.T46I and p.P56S missense mutations that cause ALS are located in the MSP. On the other hand, the p.ΔS160 deletion, located on the CC is common and does not cause ALS. Additionally, another study detected a missense variant p.D130E that showed no prevalence in cases (51) and falls between the MSP and the CC domains. Mutations in the MSP could be the leading cause for the dysfunction in the motor neurons. Nevertheless, some evidence in *Drosophila* is starting to show that mutations in the C-terminal TM can cause neurodegeneration in the corresponding variant p.V234I in humans (52).

Our results follow the same trend. The identified *de novo* p.ΔV25 deletion is absent in the general population and is located in the MSP. The mutations p.R19L and p.L125 M identified in the IPDGC dataset, both from sporadic cases, are also in the MSP (Table 4), are not present in the general population and only the latter shows two heterozygous cases in ExAC. When moving towards the CC in the IPDGC dataset, we find the frequent p.D130E and p.ΔS160 variants mentioned above, and two extra missense mutations (p.M170I and p.R184Q) with higher frequencies in the general population.

We found the *VAPB de novo* variant in a trio with two additional *de novo* variants in *COL12A1* (p.R754Q) and *RUNDC3A* (p.C108F). *COL12A1* was associated with myopathy (53) and the only association to a neurodegenerative disease is its decreased amount in the cervical spinal cord of patients with ALS (54). *COL12A1* presented 96 variants in the IPDGC dataset (see Supplementary Material, Table S3). In contrast, *RUNDC3A* presented five mutations, three of them rare and observed only in patients. Due to the fact that this trio presented three confirmed *de novo* variants, it is not possible to say with certainty, which, if any, is associated with the disease. However the fact that *VAPB* directly interacts with known PD genes strongly argues in favour of the involvement of this gene.

The results at *VAPB* and *PTEN*, which have both been shown to be involved in disparate phenotypes, fit nicely with the growing body of evidence suggesting that pleiotropic events are a greatly underappreciated event in human disease (55).

For *ASNA1*, we found one *de novo* missense mutation p.L303V in one of the trios not present in any database. High impact variants in this gene seem rare, with mainly missense variants present in ExAC and only three frameshifts (1 allele each) and one stop-gain mutations. In the IPDGC dataset, we see a similar trend with only seven rare variants. The missense variants in IPDGC (Table 4) were identified in patients presenting with positive familial history of PD (variants: p.L61V and p.R65C); and early-onset sporadic PD (variant p.L208V). *ASNA1* is known to have a function in transporting proteins as a part of the transmembrane recognition complex (TRC). Interestingly, *ASNA1* has been shown to bind to *VAPB* (49), although a role in human neurodegenerative diseases has still to be investigated.

In conclusion, we have identified *de novo* mutations in early-onset, sporadic PD cases. We have used publicly available population genetic data and our in-house dataset of exome-sequenced PD cases and controls to replicate and confirm our findings. From our list of confirmed *de novo* mutations, three genes are particularly interesting, as they have been reported to interact with known PD genes and present rare, case-specific variants in our large cohort of PD cases.

It is plausible that some of the apparently sporadic PD cases are due to recessive or compound heterozygous mutations in genes yet to be associated with disease; however we did not identify any better recessive candidates in these trios.

The present study has some shortcomings that we are not able to immediately address. The sample size is small; unfortunately it is very difficult to identify PD cases with both parents available for genetic analyses. The methodology used, although appropriate and in line with what has been previously published for *de novo* mutations in disease, misses a significant amount of genetic variability, particularly in the form of non-coding variants. Additionally, apparently *de novo* variants can actually be inherited from a parent that has low-level mosaicism for that variant. This is a difficult issue to address [as recently shown by Acuna-Hidalgo and colleagues (56)] and would require very high sequencing coverage, perhaps even of multiple tissues, in all individuals and is thus beyond the scope of the present work. In addition, definitive proof of pathological significance for PD can only be achieved by one of the following: identifying the same mutation in complete segregation in large families, identifying multiple families with even limited segregation of the same variants or functional studies of the protein harbouring the mutations showing altered function.

On the other hand, we have been able to screen, in an unbiased manner, a cohort of over 1200 PD cases and 400 controls for mutations in all of the genes identified in our trio study, having confirmed the presence of rare, case-specific mutations in the same genes.

*De novo* mutations may be a vastly underappreciated cause of apparently sporadic forms of adult-onset disease, such as PD. We show *de novo* mutations in genes, which are known to interact with known PD-causing genes, and we suggest these may be responsible for the disease in these cases.

## Material and Methods

We performed whole-exome sequencing in 21 full trios. Criteria for inclusion of cases were based on age at onset (<40 years), typical presentation of PD with negative family history, absence of pathogenic mutations in any of the known PD genes and availability of both parents for genetic studies. All cases included are of European descent.

Exomes for the 21 trios were captured using Illumina's Nextera Rapid Capture according to the manufacturer's recommendations. Indexed and pooled libraries were then sequenced on Illumina's HiSeq2000 (100 bp, paired-end) to a mean target coverage of 30×. Reads were mapped to GRCh37 using bwa-mem (0.7.12) and followed GATK Best Practices for v3 (57). Briefly, this consisted of flagging duplicate reads, realignment around indels, base recalibration and variant calling on all trios simultaneously using the HaplotypeCaller tool. Variant qualities were then recalibrated as described in DePristo *et al*. (58). After obtaining a high quality variant set, we performed a genotype-refinement protocol, as described in (http://gatkforums.broadinstitute.org/discussion/4723/genotype-refinement-workflow). This protocol uses population and pedigree priors to improve estimates of genotype likelihoods, providing higher quality variant calls.

To confirm all relationships and identify unreported familial relationships, we performed identity-by-descent (IBD) estimation for all pairs of individuals. The proportion of IBD was obtained with PLINK (59) and varied between 0.35–0.45 for the trio pairs of each parent with the descendant, and equal to zero between parents and all the remaining pairs. The lower end of the spectrum of IBD values is due to lower variant qualities in some samples.

We used three publicly available resources to extract genome-wide variant frequency data: the Exome Sequencing Project (ESP6500) (60), the 1000 Genomes Project (Oct 2014) (61) and the ExAC (62).

Sanger sequencing was used to confirm the presence of all *de novo* mutations identified by whole-exome sequencing. Primers designed for this validation are shown in the Supplementary Material (see Supplementary Material, Table S4; PCR conditions used are available upon request). Table 1 shows each of the transcripts used as basis for annotation of the variants identified.

Protein–protein interactions and functional protein associations were defined with STRING v10 (63). The input consisted of a list of known PD genes (64), and genes containing validated *de novo* variants. We considered total scores above 0.400 (medium confidence) that correspond to the combination of four different scores: co-expression, experimental, knowledge and text-mining. The total number of interactions between proteins corresponding to genes containing *de novo* variants and proteins corresponding to known PD genes was calculated, as was the number of such interactions involving the protein corresponding to *de novo* gene in turn. The significance of these quantities was assessed by randomly positioning the proteins corresponding to *de novo* and known PD genes on the interaction map such that each protein is mapped to a protein with the same total number of interactions (to avoid potential bias caused by the genes of interest having large numbers of interactions, thus being likely to interact with each other by chance). The number of interactions between each *de novo* protein and PD proteins, and the total number of such interactions, was calculated on the randomized map and compared with that observed in the actual data. This process was repeated 1 000 000 times to obtain *P*-values. Correction for multiple testing of proteins was performed by comparing the maximum number of interactions for any single protein in each simulated dataset to the number of interactions observed for each protein in the real data. A similar procedure was used to test for an excess of interactions between the proteins corresponding to known PD genes. It should be noted that throughout the text, interactions are defined as per STRING's definitions and in many cases these are only predicted, without experimental evidence.

Finally, we extracted information for genes of interest from in-house data, produced within the IPDGC. This dataset consisted of a total of 1715 samples exome-sequenced with 1243 of diagnosed PD cases and 472 controls. Details from this dataset have been previously published (65).

## Supplementary Material

Supplementary Material is available at *HMG* online.

## Funding

Funding to pay the Open Access publication charges for this article was provided by the Wellcome Trust and the Medical Research Council.

## Supplementary Material

Supplementary Data
