# Supplementary material for: A systematic screening to identify de novo mutations causing sporadic early-onset Parkinson's disease
Source: Hum Mol Genet. 2015 Sep 11;24(23):6711–20. doi: 10.1093/hmg/ddv376 (PMC4634375; doi:10.1093/hmg/ddv376)
Supplement: Supplementary Data [file supp_24_23_6711__index.html]

A systematic screening to identify de novo mutations causing sporadic early-onset Parkinson's disease — A systematic screening to identify de novo mutations causing sporadic early-onset Parkinson's disease — Supplementary Data 

# A systematic screening to identify *de novo* mutations causing sporadic early-onset Parkinson's disease

## Supplementary Data

Supplementary Data

- Supplementary File 1 - docx file
- Supplementary File 2 - docx file
- Supplementary Figures - docx file
- Supplementary Tables - xlsx file
