# Supplementary material for: A systematic screening to identify de novo mutations causing sporadic early-onset Parkinson's disease
Source: Hum Mol Genet. 2015 Sep 11;24(23):6711–20. doi: 10.1093/hmg/ddv376 (PMC4634375; doi:10.1093/hmg/ddv376)
Supplement: Supplementary Data [file supp_ddv376_ddv376supp_file1.docx]

ACKNOWLEDGMENTS

We would like to thank all of the subjects who donated their time and biological samples to be a part of this study.

This work was supported in part by the Intramural Research Programs of the National Institute of Neurological Disorders and Stroke (NINDS), the National Institute on Aging (NIA), and the National Institute of Environmental Health Sciences both part of the National Institutes of Health, Department of Health and Human Services; project numbers Z01-AG000949-02 and Z01-ES101986. In addition this work was supported by the Department of Defense (award W81XWH-09-2-0128), and The Michael J Fox Foundation for Parkinson’s Research. This work was supported by National Institutes of Health grants R01NS037167, R01CA141668, P50NS071674, American Parkinson Disease Association (APDA); Barnes Jewish Hospital Foundation; Greater St Louis Chapter of the APDA; Hersenstichting Nederland; Neuroscience Campus Amsterdam; and the section of medical genomics, the Prinses Beatrix Fonds. The KORA (Cooperative Research in the Region of Augsburg) research platform was started and financed by the Forschungszentrum für Umwelt und Gesundheit, which is funded by the German Federal Ministry of Education, Science, Research, and Technology and by the State of Bavaria. This study was also funded by the German National Genome Network (NGFNplus number 01GS08134, German Ministry for Education and Research); by the German Federal Ministry of Education and Research (NGFN 01GR0468, PopGen); and 01EW0908 in the frame of ERA-NET NEURON and Helmholtz Alliance Mental Health in an Ageing Society (HA-215), which was funded by the Initiative and Networking Fund of the Helmholtz Association. The French GWAS work was supported by the French National Agency of Research (ANR-08-MNP-012). This study was also funded by France-Parkinson Association, the French program “Investissements d’avenir” funding (ANR-10-IAIHU-06) and a grant from Assistance Publique-Hôpitaux de Paris (PHRC, AOR-08010) for the French clinical data. This study was also sponsored by the Landspitali University Hospital Research Fund (grant to SSv); Icelandic Research Council (grant to SSv); and European Community Framework Programme 7, People Programme, and IAPP on novel genetic and phenotypic markers of Parkinson’s disease and Essential Tremor (MarkMD), contract number PIAP-GA-2008-230596 MarkMD (to HP and JHu). This study utilized the high-performance computational capabilities of the Biowulf Linux cluster at the National Institutes of Health, Bethesda, Md. (http://biowulf.nih.gov), and DNA panels, samples, and clinical data from the National Institute of Neurological Disorders and Stroke Human Genetics Resource Center DNA and Cell Line Repository. People who contributed samples are acknowledged in descriptions of every panel on the repository website. We thank the French Parkinson’s Disease Genetics Study Group and the Drug Interaction with genes (DIGPD) study group: Y Agid, M Anheim, A-M Bonnet, M Borg, A Brice, E Broussolle, J-C Corvol, P Damier, A Destée, A Dürr, F Durif, A Elbaz, D Grabil, S Klebe, P. Krack, E Lohmann, L. Lacomblez, M Martinez, V Mesnage, P Pollak, O Rascol, F Tison, C Tranchant, M Vérin, F Viallet, and M Vidailhet. We also thank the members of the French 3C Consortium: A Alpérovitch, C Berr, C Tzourio, and P Amouyel for allowing us to use part of the 3C cohort, and D Zelenika for support in generating the genome-wide molecular data. We thank P Tienari (Molecular Neurology Programme, Biomedicum, University of Helsinki), T Peuralinna (Department of Neurology, Helsinki University Central Hospital), L Myllykangas (Folkhalsan Institute of Genetics and Department of Pathology, University of Helsinki), and R Sulkava (Department of Public Health and General Practice Division of Geriatrics, University of Eastern Finland) for the Finnish controls (Vantaa85+ GWAS data). We used genome-wide association data generated by the Wellcome Trust Case-Control Consortium 2 (WTCCC2) from UK patients with Parkinson’s disease and UK control individuals from the 1958 Birth Cohort and National Blood Service. Genotyping of UK replication cases on ImmunoChip was part of the WTCCC2 project, which was funded by the Wellcome Trust (083948/Z/07/Z). UK population control data was made available through WTCCC1. This study was supported by the Medical Research Council and Wellcome Trust disease centre (grant WT089698/Z/09/Z to NW, JHa, and ASc). As with previous IPDGC efforts, this study makes use of data generated by the Wellcome Trust Case-Control Consortium. A full list of the investigators who contributed to the generation of the data is available from www.wtccc.org.uk. Funding for the project was provided by the Wellcome Trust under award 076113, 085475 and 090355. This study was also supported by Parkinson’s UK (grants 8047 and J-0804) and the Medical Research Council (G0700943). We thank Jeffrey Barrett for assistance with the design of the ImmunoChip. DNA extraction work that was done in the UK was undertaken at University College London Hospitals, University College London, who received a proportion of funding from the Department of Health’s National Institute for Health Research Biomedical Research Centres funding. This study was supported in part by the Wellcome Trust/Medical Research Council Joint Call in Neurodegeneration award (WT089698) to the Parkinson’s Disease Consortium (UKPDC), whose members are from the UCL Institute of Neurology, University of Sheffield, and the Medical Research Council Protein Phosphorylation Unit at the University of Dundee.

MEMBERS AND AFFILIATIONS

IPDGC consortium members and affiliations: Mike A Nalls (Laboratory of Neurogenetics, National Institute on Aging, National Institutes of Health, Bethesda, MD, USA), Vincent Plagnol (UCL Genetics Institute, London, UK), Dena G Hernandez (Laboratory of Neurogenetics, National Institute on Aging; and Department of Molecular Neuroscience, UCL Institute of Neurology, London, UK), Manu Sharma (Department for Neurodegenerative Diseases, Hertie Institute for Clinical Brain Research, University of Tübingen, and DZNE, German Center for Neurodegenerative Diseases, Tübingen, Germany), Una-Marie Sheerin (Department of Molecular Neuroscience, UCL Institute of Neurology), Mohamad Saad (INSERM U563, CPTP, Toulouse, France; and Paul Sabatier University, Toulouse, France), Javier Simón-Sánchez (Department of Clinical Genetics, Section of Medical Genomics, VU University Medical Centre, Amsterdam, Netherlands), Claudia Schulte (Department for Neurodegenerative Diseases, Hertie Institute for Clinical Brain Research), Suzanne Lesage (INSERM, UMR_S975 [ formerly UMR_S679], Paris, France; Université Pierre et Marie Curie-Paris, Centre de Recherche de l’Institut du Cerveau et de la Moelle épinière, Paris, France; and CNRS, Paris, France), Sigurlaug Sveinbjörnsdóttir (Department of Neurology, Landspítali University Hospital, Reykjavík, Iceland; Department of Neurology, MEHT Broomfield Hospital, Chelmsford, Essex, UK; and Queen Mary College, University of London, London, UK), Sampath Arepalli (Laboratory of Neurogenetics, National Institute on Aging), Roger Barker (Department of Neurology, Addenbrooke’s Hospital, University of Cambridge, Cambridge, UK), Yoav Ben-Shlomo (School of Social and Community Medicine, University of Bristol), Henk W Berendse (Department of Neurology and Alzheimer Center, VU University Medical Center), Daniela Berg (Department for Neurodegenerative Diseases, Hertie Institute for Clinical Brain Research and DZNE, German Center for Neurodegenerative diseases), Kailash Bhatia (Department of Motor Neuroscience, UCL Institute of Neurology), Rob M A de Bie (Department of Neurology, Academic Medical Center, University of Amsterdam, Amsterdam, Netherlands), Alessandro Biffi (Center for Human Genetic Research and Department of Neurology, Massachusetts General Hospital, Boston, MA, USA; and Program in Medical and Population Genetics, Broad Institute, Cambridge, MA, USA), Bas Bloem (Department of Neurology, Radboud University Nijmegen Medical Centre, Nijmegen, Netherlands), Zoltan Bochdanovits (Department of Clinical Genetics, Section of Medical Genomics, VU University Medical Centre), Michael Bonin (Department of Medical Genetics, Institute of Human Genetics, University of Tübingen, Tübingen, Germany), Jose M Bras (Department of Molecular Neuroscience, UCL Institute of Neurology), Kathrin Brockmann (Department for Neurodegenerative Diseases, Hertie Institute for Clinical Brain Research and DZNE, German Center for Neurodegenerative diseases), Janet Brooks (Laboratory of Neurogenetics, National Institute on Aging), David J Burn (Newcastle University Clinical Ageing Research Unit, Campus for Ageing and Vitality, Newcastle upon Tyne, UK), Gavin Charlesworth (Department of Molecular Neuroscience, UCL Institute of Neurology), Honglei Chen (Epidemiology Branch, National Institute of Environmental Health Sciences, National Institutes of Health, NC, USA), Patrick F Chinnery (Neurology M4104, The Medical School, Framlington Place, Newcastle upon Tyne, UK), Sean Chong (Laboratory of Neurogenetics, National Institute on Aging), Carl E Clarke (School of Clinical and Experimental Medicine, University of Birmingham, Birmingham, UK; and Department of Neurology, City Hospital, Sandwell and West Birmingham Hospitals NHS Trust, Birmingham, UK), Mark R Cookson (Laboratory of Neurogenetics, National Institute on Aging), J Mark Cooper (Department of Clinical Neurosciences, UCL Institute of Neurology), Jean Christophe Corvol (INSERM, UMR_S975; Université Pierre et Marie Curie-Paris; CNRS; and INSERM CIC-9503, Hôpital Pitié-Salpêtrière, Paris, France), Carl Counsell (University of Aberdeen, Division of Applied Health Sciences, Population Health Section, Aberdeen, UK), Philippe Damier (CHU Nantes, CIC0004, Service de Neurologie, Nantes, France), Jean-François Dartigues (INSERM U897, Université Victor Segalen, Bordeaux, France), Panos Deloukas (Wellcome Trust Sanger Institute, Wellcome Trust Genome Campus, Cambridge, UK), Günther Deuschl (Klinik für Neurologie, Universitätsklinikum Schleswig-Holstein, Campus Kiel, Christian-Albrechts-Universität Kiel, Kiel, Germany), David T Dexter (Parkinson’s Disease Research Group, Faculty of Medicine, Imperial College London, London, UK), Karin D van Dijk (Department of Neurology and Alzheimer Center, VU University Medical Center), Allissa Dillman (Laboratory of Neurogenetics, National Institute on Aging), Frank Durif (Service de Neurologie, Hôpital Gabriel Montpied, Clermont-Ferrand, France), Alexandra Dürr (INSERM, UMR_S975; Université Pierre et Marie Curie-Paris; CNRS; and AP-HP, Pitié-Salpêtrière Hospital), Sarah Edkins (Wellcome Trust Sanger Institute), Jonathan R Evans (Cambridge Centre for Brain Repair, Cambridge, UK), Thomas Foltynie (UCL Institute of Neurology), Jing Dong (Epidemiology Branch, National Institute of Environmental Health Sciences), Michelle Gardner (Department of Molecular Neuroscience, UCL Institute of Neurology), J Raphael Gibbs (Laboratory of Neurogenetics, National Institute on Aging; and Department of Molecular Neuroscience, UCL Institute of Neurology), Alison Goate (Department of Psychiatry, Department of Neurology, Washington University School of Medicine, MI, USA), Emma Gray (Wellcome Trust Sanger Institute), Rita Guerreiro (Department of Molecular Neuroscience, UCL Institute of Neurology), Clare Harris (University of Aberdeen), Jacobus J van Hilten (Department of Neurology, Leiden University Medical Center, Leiden, Netherlands), Albert Hofman (Department of Epidemiology, Erasmus University Medical Center, Rotterdam, Netherlands), Albert Hollenbeck (AARP, Washington DC, USA), Janice Holton (Queen Square Brain Bank for Neurological Disorders, UCL Institute of Neurology), Michele Hu (Department of Clinical Neurology, John Radcliffe Hospital, Oxford, UK), Xuemei Huang (Departments of Neurology, Radiology, Neurosurgery, Pharmacology, Kinesiology, and Bioengineering, Pennsylvania State University– Milton S Hershey Medical Center, Hershey, PA, USA), Isabel Wurster (Department for Neurodegenerative Diseases, Hertie Institute for Clinical Brain Research and German Center for Neurodegenerative diseases), Walter Mätzler (Department for Neurodegenerative Diseases, Hertie Institute for Clinical Brain Research and German Center for Neurodegenerative diseases), Gavin Hudson (Neurology M4104, The Medical School, Newcastle upon Tyne, UK), Sarah E Hunt (Wellcome Trust Sanger Institute), Johanna Huttenlocher (deCODE genetics), Thomas Illig (Institute of Epidemiology, Helmholtz Zentrum München, German Research Centre for Environmental Health, Neuherberg, Germany), Pálmi V Jónsson (Department of Geriatrics, Landspítali University Hospital, Reykjavík, Iceland), Jean-Charles Lambert (INSERM U744, Lille, France; and Institut Pasteur de Lille, Université de Lille Nord, Lille, France), Cordelia Langford (Cambridge Centre for Brain Repair), Andrew Lees (Queen Square Brain Bank for Neurological Disorders), Peter Lichtner (Institute of Human Genetics, Helmholtz Zentrum München, German Research Centre for Environmental Health, Neuherberg, Germany), Patricia Limousin (Institute of Neurology, Sobell Department, Unit of Functional Neurosurgery, London, UK), Grisel Lopez (Section on Molecular Neurogenetics, Medical Genetics Branch, NHGRI, National Institutes of Health), Delia Lorenz (Klinik für Neurologie, Universitätsklinikum Schleswig-Holstein), Alisdair McNeill (Department of Clinical Neurosciences, UCL Institute of Neurology), Catriona Moorby (School of Clinical and Experimental Medicine, University of Birmingham), Matthew Moore (Laboratory of Neurogenetics, National Institute on Aging), Huw R Morris (MRC Centre for Neuropsychiatric Genetics and Genomics, Cardiff University School of Medicine, Cardiff, UK), Karen E Morrison (School of Clinical and Experimental Medicine, University of Birmingham; and Neurosciences Department, Queen Elizabeth Hospital, University Hospitals Birmingham NHS Foundation Trust, Birmingham, UK), Ese Mudanohwo (Neurogenetics Unit, UCL Institute of Neurology and National Hospital for Neurology and Neurosurgery), Sean S O’Sullivan (Queen Square Brain Bank for Neurological Disorders), Justin Pearson (MRC Centre for Neuropsychiatric Genetics and Genomics), Joel S Perlmutter (Department of Neurology, Radiology, and Neurobiology at Washington University, St Louis), Hjörvar Pétursson (deCODE genetics; and Department of Medical Genetics, Institute of Human Genetics, University of Tübingen), Pierre Pollak (Service de Neurologie, CHU de Grenoble, Grenoble, France), Bart Post (Department of Neurology, Radboud University Nijmegen Medical Centre), Simon Potter (Wellcome Trust Sanger Institute), Bernard Ravina (Translational Neurology, Biogen Idec, MA, USA), Tamas Revesz (Queen Square Brain Bank for Neurological Disorders), Olaf Riess (Department of Medical Genetics, Institute of Human Genetics, University of Tübingen), Fernando Rivadeneira (Departments of Epidemiology and Internal Medicine, Erasmus University Medical Center), Patrizia Rizzu (Department of Clinical Genetics, Section of Medical Genomics, VU University Medical Centre), Mina Ryten (Department of Molecular Neuroscience, UCL Institute of Neurology), Stephen Sawcer (University of Cambridge, Department of Clinical Neurosciences, Addenbrooke’s hospital, Cambridge, UK), Anthony Schapira (Department of Clinical Neurosciences, UCL Institute of Neurology), Hans Scheffer (Department of Human Genetics, Radboud University Nijmegen Medical Centre, Nijmegen, Netherlands), Karen Shaw (Queen Square Brain Bank for Neurological Disorders), Ira Shoulson (Department of Neurology, University of Rochester, Rochester, NY, USA), Ellen Sidransky (Section on Molecular Neurogenetics, Medical Genetics Branch, NHGRI), Colin Smith (Department of Pathology, University of Edinburgh, Edinburgh, UK), Chris C A Spencer (Wellcome Trust Centre for Human Genetics, Oxford, UK), Hreinn Stefánsson (deCODE genetics), Francesco Bettella (deCODE genetics), Joanna D Stockton (School of Clinical and Experimental Medicine), Amy Strange (Wellcome Trust Centre for Human Genetics), Kevin Talbot (University of Oxford, Department of Clinical Neurology, John Radcliffe Hospital, Oxford, UK), Carlie M Tanner (Clinical Research Department, The Parkinson’s Institute and Clinical Center, Sunnyvale, CA, USA), Avazeh Tashakkori-Ghanbaria (Wellcome Trust Sanger Institute), François Tison (Service de Neurologie, Hôpital Haut-Lévêque, Pessac, France), Daniah Trabzuni (Department of Molecular Neuroscience, UCL Institute of Neurology), Bryan J Traynor (Laboratory of Neurogenetics, National Institute on Aging), André G Uitterlinden (Departments of Epidemiology and Internal Medicine, Erasmus University Medical Center), Daan Velseboer (Department of Neurology, Academic Medical Center), Marie Vidailhet (INSERM, UMR_S975, Université Pierre et Marie Curie-Paris, CNRS, UMR 7225), Robert Walker (Department of Pathology, University of Edinburgh), Bart van de Warrenburg (Department of Neurology, Radboud University Nijmegen Medical Centre), Mirdhu Wickremaratchi (Department of Neurology, Cardiff University, Cardiff, UK), Nigel Williams (MRC Centre for Neuropsychiatric Genetics and Genomics), Caroline H Williams-Gray (Department of Neurology, Addenbrooke’s Hospital), Sophie Winder-Rhodes (Department of Psychiatry and Medical Research Council and Wellcome Trust Behavioural and Clinical Neurosciences Institute, University of Cambridge), Kári Stefánsson (deCODE genetics), Maria Martinez (INSERM UMR 1043; and Paul Sabatier University), Nicholas W Wood (UCL Genetics Institute; and Department of Molecular Neuroscience, UCL Institute of Neurology), John Hardy (Department of Molecular Neuroscience, UCL Institute of Neurology), Peter Heutink (Department of Clinical Genetics, Section of Medical Genomics, VU University Medical Centre), Alexis Brice (INSERM, UMR_S975, Université Pierre et Marie Curie-Paris, CNRS, UMR 7225, AP-HP, Pitié-Salpêtrière Hospital), Thomas Gasser (Department for Neurodegenerative Diseases, Hertie Institute for Clinical Brain Research, and DZNE, German Center for Neurodegenerative Diseases), Andrew B Singleton (Laboratory of Neurogenetics, National Institute on Aging).
