# Supplementary material for: A systematic screening to identify de novo mutations causing sporadic early-onset Parkinson's disease
Source: Hum Mol Genet. 2015 Sep 11;24(23):6711–20. doi: 10.1093/hmg/ddv376 (PMC4634375; doi:10.1093/hmg/ddv376)
Supplement: Supplementary Data [file supp_ddv376_ddv376supp_file2.docx]

**IPDGC consortium members and affiliations:** Mike A Nalls (Laboratory of Neurogenetics, National Institute on Aging, National Institutes of Health, Bethesda, MD, USA), Vincent Plagnol (UCL Genetics Institute, London, UK), Dena G Hernandez (Laboratory of Neurogenetics, National Institute on Aging; and Department of Molecular Neuroscience, UCL Institute of Neurology, London, UK), Manu Sharma (Department for Neurodegenerative Diseases, Hertie Institute for Clinical Brain Research, University of Tübingen, and DZNE, German Center for Neurodegenerative Diseases, Tübingen, Germany), Una-Marie Sheerin (Department of Molecular Neuroscience, UCL Institute of Neurology), Mohamad Saad (INSERM U563, CPTP, Toulouse, France; and Paul Sabatier University, Toulouse, France), Javier Simón-Sánchez (Department of Clinical Genetics, Section of Medical Genomics, VU University Medical Centre, Amsterdam, Netherlands), Claudia Schulte (Department for Neurodegenerative Diseases, Hertie Institute for Clinical Brain Research), Suzanne Lesage (INSERM, UMR_S975 [ formerly UMR_S679], Paris, France; Université Pierre et Marie Curie-Paris, Centre de Recherche de l’Institut du Cerveau et de la Moelle épinière, Paris, France; and CNRS, Paris, France), Sigurlaug Sveinbjörnsdóttir (Department of Neurology, Landspítali University Hospital, Reykjavík, Iceland; Department of Neurology, MEHT Broomfield Hospital, Chelmsford, Essex, UK; and Queen Mary College, University of London, London, UK), Sampath Arepalli (Laboratory of Neurogenetics, National Institute on Aging), Roger Barker (Department of Neurology, Addenbrooke’s Hospital, University of Cambridge, Cambridge, UK), Yoav Ben-Shlomo (School of Social and Community Medicine, University of Bristol), Henk W Berendse (Department of Neurology and Alzheimer Center, VU University Medical Center), Daniela Berg (Department for Neurodegenerative Diseases, Hertie Institute for Clinical Brain Research and DZNE, German Center for Neurodegenerative diseases), Kailash Bhatia (Department of Motor Neuroscience, UCL Institute of Neurology), Rob M A de Bie (Department of Neurology, Academic Medical Center, University of Amsterdam, Amsterdam, Netherlands), Alessandro Biffi (Center for Human Genetic Research and Department of Neurology, Massachusetts General Hospital, Boston, MA, USA; and Program in Medical and Population Genetics, Broad Institute, Cambridge, MA, USA), Bas Bloem (Department of Neurology, Radboud University Nijmegen Medical Centre, Nijmegen, Netherlands), Zoltan Bochdanovits (Department of Clinical Genetics, Section of Medical Genomics, VU University Medical Centre), Michael Bonin (Department of Medical Genetics, Institute of Human Genetics, University of Tübingen, Tübingen, Germany), Jose M Bras (Department of Molecular Neuroscience, UCL Institute of Neurology), Kathrin Brockmann (Department for Neurodegenerative Diseases, Hertie Institute for Clinical Brain Research and DZNE, German Center for Neurodegenerative diseases), Janet Brooks (Laboratory of Neurogenetics, National Institute on Aging), David J Burn (Newcastle University Clinical Ageing Research Unit, Campus for Ageing and Vitality, Newcastle upon Tyne, UK), Elisa Majounie (Laboratory of Neurogenetics, National Institute on Aging), Gavin Charlesworth (Department of Molecular Neuroscience, UCL Institute of Neurology), Codrin Lungu (National Institutes of Health Parkinson Clinic, NINDS, National Institutes of Health), Honglei Chen (Epidemiology Branch, National Institute of Environmental Health Sciences, National Institutes of Health, NC, USA), Patrick F Chinnery (Neurology M4104, The Medical School, Framlington Place, Newcastle upon Tyne, UK), Sean Chong (Laboratory of Neurogenetics, National Institute on Aging), Carl E Clarke (School of Clinical and Experimental Medicine, University of Birmingham, Birmingham, UK; and Department of Neurology, City Hospital, Sandwell and West Birmingham Hospitals NHS Trust, Birmingham, UK), Mark R Cookson (Laboratory of Neurogenetics, National Institute on Aging), J Mark Cooper (Department of Clinical Neurosciences, UCL Institute of Neurology), Jean Christophe Corvol (INSERM, UMR_S975; Université Pierre et Marie Curie-Paris; CNRS; and INSERM CIC-9503, Hôpital Pitié-Salpêtrière, Paris, France), Carl Counsell (University of Aberdeen, Division of Applied Health Sciences, Population Health Section, Aberdeen, UK), Philippe Damier (CHU Nantes, CIC0004, Service de Neurologie, Nantes, France), Jean-François Dartigues (INSERM U897, Université Victor Segalen, Bordeaux, France), Panos Deloukas (Wellcome Trust Sanger Institute, Wellcome Trust Genome Campus, Cambridge, UK), Günther Deuschl (Klinik für Neurologie, Universitätsklinikum Schleswig-Holstein, Campus Kiel, Christian-Albrechts-Universität Kiel, Kiel, Germany), David T Dexter (Parkinson’s Disease Research Group, Faculty of Medicine, Imperial College London, London, UK), Karin D van Dijk (Department of Neurology and Alzheimer Center, VU University Medical Center), Allissa Dillman (Laboratory of Neurogenetics, National Institute on Aging), Frank Durif (Service de Neurologie, Hôpital Gabriel Montpied, Clermont-Ferrand, France), Alexandra Dürr (INSERM, UMR_S975; Université Pierre et Marie Curie-Paris; CNRS; and AP-HP, Pitié-Salpêtrière Hospital), Sarah Edkins (Wellcome Trust Sanger Institute), Jonathan R Evans (Cambridge Centre for Brain Repair, Cambridge, UK), Thomas Foltynie (UCL Institute of Neurology), Jing Dong (Epidemiology Branch, National Institute of Environmental Health Sciences), Michelle Gardner (Department of Molecular Neuroscience, UCL Institute of Neurology), J Raphael Gibbs (Laboratory of Neurogenetics, National Institute on Aging; and Department of Molecular Neuroscience, UCL Institute of Neurology), Alison Goate (Department of Psychiatry, Department of Neurology, Washington University School of Medicine, MI, USA), Emma Gray (Wellcome Trust Sanger Institute), Rita Guerreiro (Department of Molecular Neuroscience, UCL Institute of Neurology), Clare Harris (University of Aberdeen), Jacobus J van Hilten (Department of Neurology, Leiden University Medical Center, Leiden, Netherlands), Albert Hofman (Department of Epidemiology, Erasmus University Medical Center, Rotterdam, Netherlands), Albert Hollenbeck (AARP, Washington DC, USA), Janice Holton (Queen Square Brain Bank for Neurological Disorders, UCL Institute of Neurology), Michele Hu (Department of Clinical Neurology, John Radcliffe Hospital, Oxford, UK), Xuemei Huang (Departments of Neurology, Radiology, Neurosurgery, Pharmacology, Kinesiology, and Bioengineering, Pennsylvania State University– Milton S Hershey Medical Center, Hershey, PA, USA), Isabel Wurster (Department for Neurodegenerative Diseases, Hertie Institute for Clinical Brain Research and German Center for Neurodegenerative diseases), Walter Mätzler (Department for Neurodegenerative Diseases, Hertie Institute for Clinical Brain Research and German Center for Neurodegenerative diseases), Gavin Hudson (Neurology M4104, The Medical School, Newcastle upon Tyne, UK), Sarah E Hunt (Wellcome Trust Sanger Institute), Johanna Huttenlocher (deCODE genetics), Thomas Illig (Institute of Epidemiology, Helmholtz Zentrum München, German Research Centre for Environmental Health, Neuherberg, Germany), Pálmi V Jónsson (Department of Geriatrics, Landspítali University Hospital, Reykjavík, Iceland), Jean-Charles Lambert (INSERM U744, Lille, France; and Institut Pasteur de Lille, Université de Lille Nord, Lille, France), Cordelia Langford (Cambridge Centre for Brain Repair), Andrew Lees (Queen Square Brain Bank for Neurological Disorders), Peter Lichtner (Institute of Human Genetics, Helmholtz Zentrum München, German Research Centre for Environmental Health, Neuherberg, Germany), Patricia Limousin (Institute of Neurology, Sobell Department, Unit of Functional Neurosurgery, London, UK), Grisel Lopez (Section on Molecular Neurogenetics, Medical Genetics Branch, NHGRI, National Institutes of Health), Delia Lorenz (Klinik für Neurologie, Universitätsklinikum Schleswig-Holstein), Codrin Lungu (National Institutes of Health Parkinson Clinic, NINDS, National Institutes of Health), Alisdair McNeill (Department of Clinical Neurosciences, UCL Institute of Neurology), Catriona Moorby (School of Clinical and Experimental Medicine, University of Birmingham), Matthew Moore (Laboratory of Neurogenetics, National Institute on Aging), Huw R Morris (National Hospital for Neurology and Neurosurgery, University College London, London, UK), Karen E Morrison (School of Clinical and Experimental Medicine, University of Birmingham; and Neurosciences Department, Queen Elizabeth Hospital, University Hospitals Birmingham NHS Foundation Trust, Birmingham, UK), Valentina Escott-Price (MRC Centre for Neuropsychiatric Genetics and Genomics, Cardiff University School of Medicine, Cardiff, UK), Ese Mudanohwo (Neurogenetics Unit, UCL Institute of Neurology and National Hospital for Neurology and Neurosurgery), Sean S O’Sullivan (Queen Square Brain Bank for Neurological Disorders), Justin Pearson (MRC Centre for Neuropsychiatric Genetics and Genomics), Joel S Perlmutter (Department of Neurology, Radiology, and Neurobiology at Washington University, St Louis), Hjörvar Pétursson (deCODE genetics; and Department of Medical Genetics, Institute of Human Genetics, University of Tübingen), Pierre Pollak (Service de Neurologie, CHU de Grenoble, Grenoble, France), Bart Post (Department of Neurology, Radboud University Nijmegen Medical Centre), Simon Potter (Wellcome Trust Sanger Institute), Bernard Ravina (Translational Neurology, Biogen Idec, MA, USA), Tamas Revesz (Queen Square Brain Bank for Neurological Disorders), Olaf Riess (Department of Medical Genetics, Institute of Human Genetics, University of Tübingen), Fernando Rivadeneira (Departments of Epidemiology and Internal Medicine, Erasmus University Medical Center), Patrizia Rizzu (Department of Clinical Genetics, Section of Medical Genomics, VU University Medical Centre), Mina Ryten (Department of Molecular Neuroscience, UCL Institute of Neurology), Stephen Sawcer (University of Cambridge, Department of Clinical Neurosciences, Addenbrooke’s hospital, Cambridge, UK), Anthony Schapira (Department of Clinical Neurosciences, UCL Institute of Neurology), Hans Scheffer (Department of Human Genetics, Radboud University Nijmegen Medical Centre, Nijmegen, Netherlands), Karen Shaw (Queen Square Brain Bank for Neurological Disorders), Ira Shoulson (Department of Neurology, University of Rochester, Rochester, NY, USA), Joshua Shulman (Baylor College of Medicine, Houston, Texas), Ellen Sidransky (Section on Molecular Neurogenetics, Medical Genetics Branch, NHGRI), Colin Smith (Department of Pathology, University of Edinburgh, Edinburgh, UK), Chris C A Spencer (Wellcome Trust Centre for Human Genetics, Oxford, UK), Hreinn Stefánsson (deCODE genetics), Francesco Bettella (deCODE genetics), Joanna D Stockton (School of Clinical and Experimental Medicine), Amy Strange (Wellcome Trust Centre for Human Genetics), Kevin Talbot (University of Oxford, Department of Clinical Neurology, John Radcliffe Hospital, Oxford, UK), Carlie M Tanner (Clinical Research Department, The Parkinson’s Institute and Clinical Center, Sunnyvale, CA, USA), Avazeh Tashakkori-Ghanbaria (Wellcome Trust Sanger Institute), François Tison (Service de Neurologie, Hôpital Haut-Lévêque, Pessac, France), Daniah Trabzuni (Department of Molecular Neuroscience, UCL Institute of Neurology), Bryan J Traynor (Laboratory of Neurogenetics, National Institute on Aging), André G Uitterlinden (Departments of Epidemiology and Internal Medicine, Erasmus University Medical Center), Daan Velseboer (Department of Neurology, Academic Medical Center), Marie Vidailhet (INSERM, UMR_S975, Université Pierre et Marie Curie-Paris, CNRS, UMR 7225), Robert Walker (Department of Pathology, University of Edinburgh), Bart van de Warrenburg (Department of Neurology, Radboud University Nijmegen Medical Centre), Mirdhu Wickremaratchi (Department of Neurology, Cardiff University, Cardiff, UK), Nigel Williams (MRC Centre for Neuropsychiatric Genetics and Genomics), Caroline H Williams-Gray (Department of Neurology, Addenbrooke’s Hospital), Sophie Winder-Rhodes (Department of Psychiatry and Medical Research Council and Wellcome Trust Behavioural and Clinical Neurosciences Institute, University of Cambridge), Kári Stefánsson (deCODE genetics), Maria Martinez (INSERM UMR 1043; and Paul Sabatier University), Nicholas W Wood (UCL Genetics Institute; and Department of Molecular Neuroscience, UCL Institute of Neurology), John Hardy (Department of Molecular Neuroscience, UCL Institute of Neurology), Peter Heutink (Department of Clinical Genetics, Section of Medical Genomics, VU University Medical Centre), Alexis Brice (INSERM, UMR_S975, Université Pierre et Marie Curie-Paris, CNRS, UMR 7225, AP-HP, Pitié-Salpêtrière Hospital), Thomas Gasser (Department for Neurodegenerative Diseases, Hertie Institute for Clinical Brain Research, and DZNE, German Center for Neurodegenerative Diseases), Andrew B Singleton (Laboratory of Neurogenetics, National Institute on Aging).
