# Supplementary material for: A systematic screening to identify de novo mutations causing sporadic early-onset Parkinson's disease
Source: Hum Mol Genet. 2015 Sep 11;24(23):6711–20. doi: 10.1093/hmg/ddv376 (PMC4634375; doi:10.1093/hmg/ddv376)
Supplement: Supplementary Data [file supp_ddv376_ddv376supp_figs.docx]

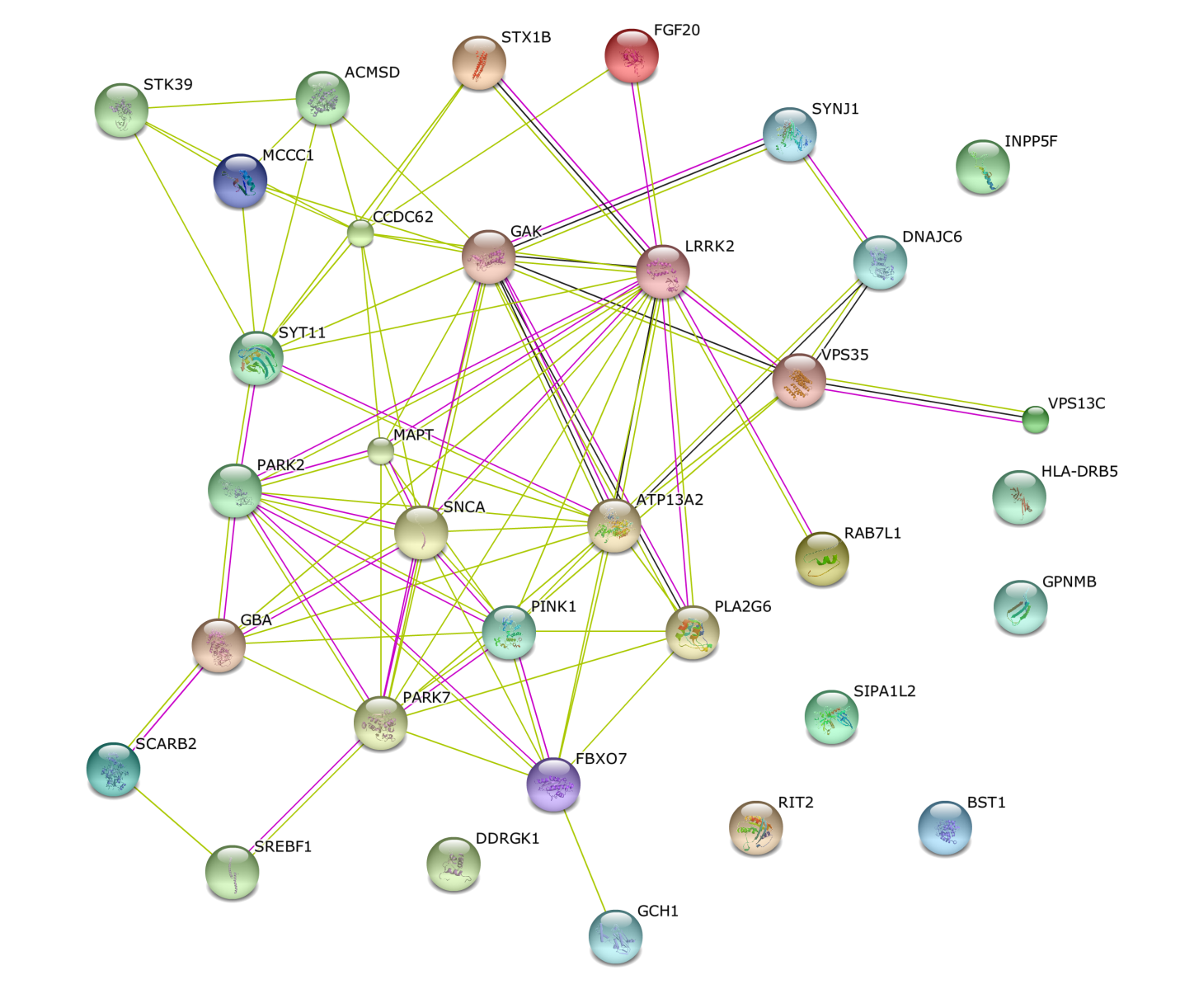


**Figure S1.** Known interaction network for PD genes.


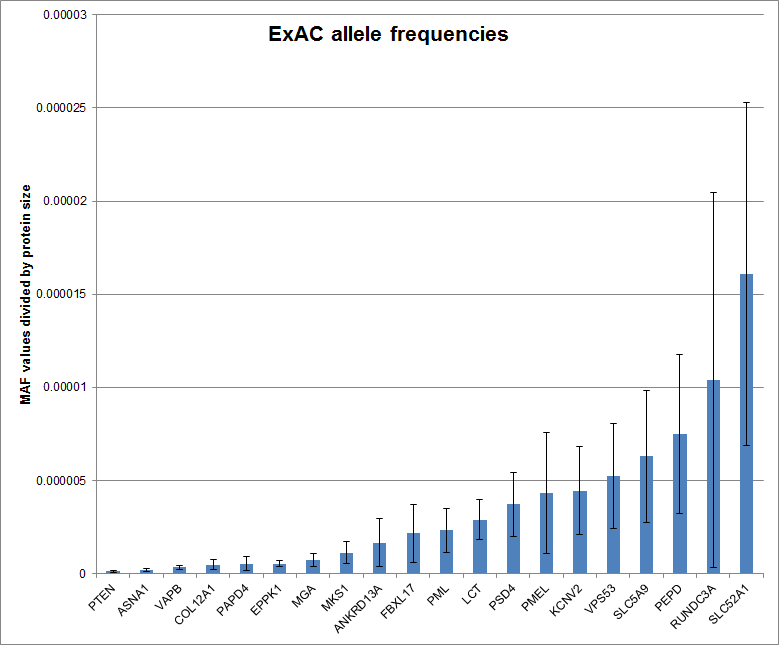


**Figure S2.** Average MAF values in ExAC for the genes studied. Averages contain the total number of mutations located on the exons. Averages are divided by the protein size (amino acid number) for each gene and the bars correspond to the standard error of the mean.
